# Posterior Extradural Spinal Tumor Extending From T3 to T6 Presenting With Paraplegia

**DOI:** 10.7759/cureus.94132

**Published:** 2025-10-08

**Authors:** Mauricio Rousseau Crespo, Isabel Yustina Espinoza Leaños, Maria Jose Guaman, Jorge Bejarano Cardenas

**Affiliations:** 1 General Medicine, Universidad Catolica Boliviana "San Pablo" Santa Cruz, Santa Cruz de la Sierra, BOL; 2 Medicine, Universidad Catolica Boliviana "San Pablo" Santa Cruz, Santa Cruz de la Sierra, BOL; 3 Neurosurgery, Hospital San Juan De Dios, Santa Cruz de la Sierra, BOL

**Keywords:** benign spinal lipoma, extradural spinal tumors, laminectomy, nerve root compression, primary spinal lymphoma, progressive dorsal pain, spinal angiolipomas extradural (seal), spinal cord compression

## Abstract

Extradural spinal tumors represent a large proportion of all spinal tumors and commonly present with spinal cord or nerve root compression. Although metastatic lesions are the most frequent, benign and less common entities may also occur. Prompt recognition is essential to prevent irreversible neurological compromise. We report the case of a 55-year-old male patient with diabetes mellitus who presented with paraplegia following three months of progressive dorsal pain and lower limb paresthesias. Neurological examination revealed flaccid paralysis of the lower limbs, sensory loss below the T5 dermatome, and sphincter dysfunction. Thoracic spine MRI demonstrated a posterior extradural mass extending from T3 to T6, compressing the spinal cord and associated with hematomyelia. The patient underwent urgent posterior decompression with wide laminectomy, complete tumor resection, and instrumented stabilization using transpedicular screws and titanium rods. Vancomycin powder was applied intraoperatively for infection prophylaxis. Postoperatively, the patient regained superficial sensation in the lower limbs and sphincter control within two months. At six months, neurological function improved to American Spinal Injury Association (ASIA) grade C, with muscle strength graded 3/5 at the hips and knees and 2/5 at the ankles according to the Medical Research Council (MRC) scale, allowing assisted ambulation through intensive physiotherapy. Histopathological examination confirmed a fibrolipoma with secondary ischemic and inflammatory changes and partial bone necrosis, without evidence of malignancy. Reporting such cases contributes to the understanding of the clinical spectrum of extradural spinal tumors and reinforces the need for prompt recognition and intervention.

## Introduction

Extradural spinal tumors are lesions that arise outside the dural sac but within the spinal canal, representing approximately 60% of all spinal tumors [1]. They typically compress the spinal cord or nerve roots, leading to variable neurological deficits depending on their size and location. Most extradural lesions are metastatic, commonly originating from primary malignancies of the lung, breast, prostate, kidney, or thyroid, or from hematologic neoplasms such as lymphoma and multiple myeloma [2]. Less frequently, benign lesions include lipomas, angiolipomas, or fibrolipomas may occur in the same compartment.

Clinically, these tumors may manifest with progressive dorsal or radicular pain, followed by sensory disturbances, motor weakness, or sphincter dysfunction as cord compression evolves. The tempo of symptom progression (gradual vs. rapid) can help distinguish benign from malignant etiologies. Magnetic resonance imaging (MRI) is the gold standard for diagnosis, as it provides superior soft tissue characterization and accurately defines the lesion’s extension and relationship to the spinal cord. In contrast, computed tomography (CT) may appear normal in early stages, which can delay diagnosis in atypical cases. Treatment typically requires urgent surgical decompression, with or without adjuvant therapy, to preserve neurological function, relieve pain, and maintain spinal stability [3].

Early recognition and prompt management are essential to prevent permanent disability. Delayed diagnosis or treatment may lead to irreversible spinal cord compression, progressive paralysis, and permanent neurological deficits, particularly when associated with rare complications such as hematomyelia, which can further exacerbate spinal cord injury due to intramedullary hemorrhage. Reporting rare or atypical cases contributes to the medical literature by highlighting diagnostic challenges, therapeutic strategies, and clinical outcomes.

## Case presentation

A 55-year-old male presented to the emergency department with paraplegia after three months of progressive, predominantly nocturnal dorsal pain followed by lower-limb paresthesias and a T5 sensory level. He had a history of well-controlled type 2 diabetes mellitus managed with metformin and no prior surgeries.

On examination, the Glasgow Coma Scale was 15/15. There was flaccid paralysis of the lower limbs with preserved trophism, absence of superficial and deep sensation below T5, and loss of sphincter control. Sacral sparing was not documented at presentation; therefore, the patient was conservatively classified as American Spinal Injury Association (ASIA) Impairment Scale (AIS) A on admission. Deep tendon reflexes in the lower limbs were depressed.

**Figure 1:**
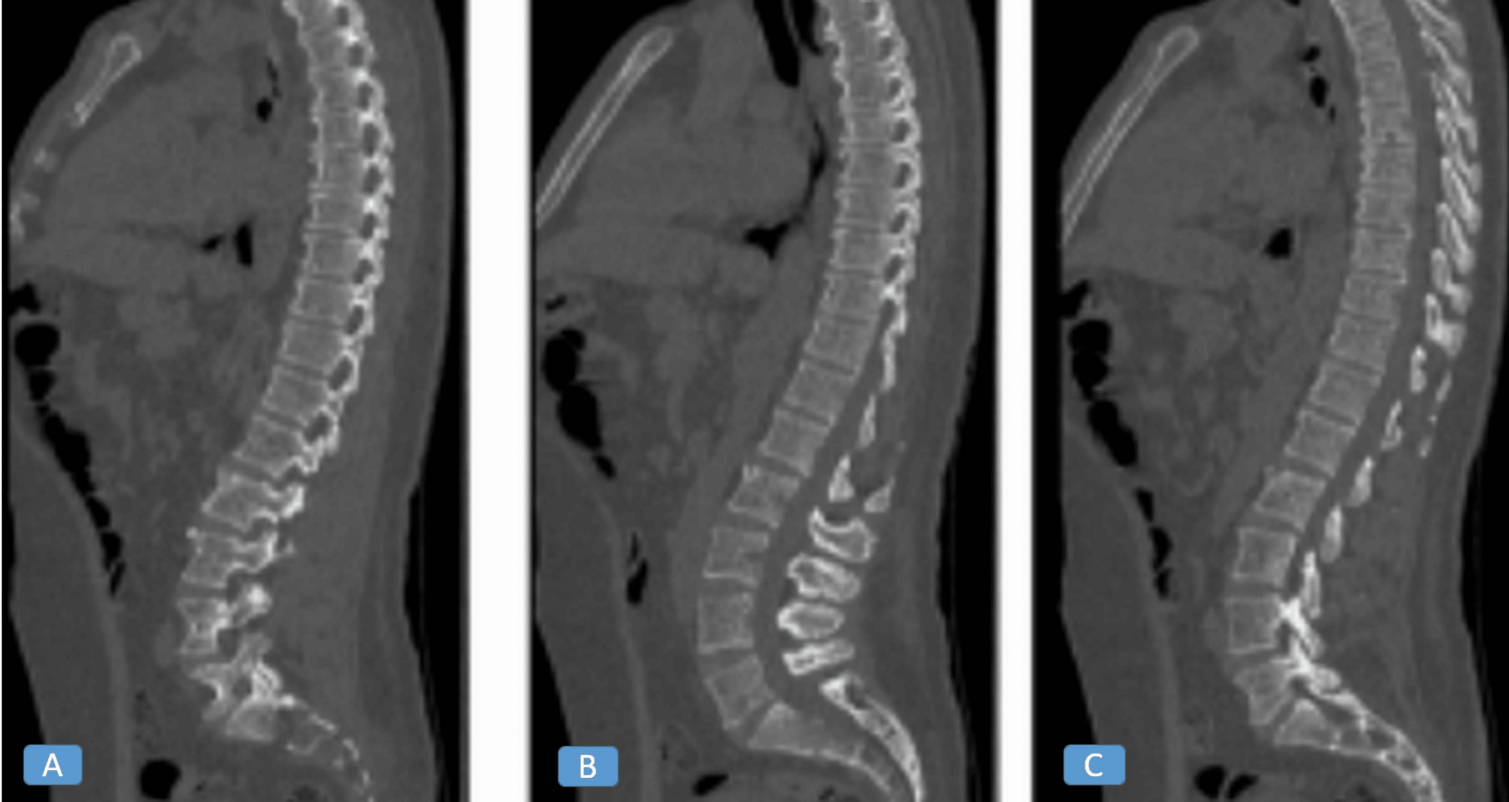
Sagittal computed tomography of the thoracolumbar spine Showing preserved vertebral alignment, normal vertebral body morphology, and a patent spinal canal without evidence of fracture, destructive lesions, or extradural masses. Reconstructions are displayed in (A) bone window, (B) soft tissue window, and (C) myelographic reconstruction.

A CT scan of the thoracolumbar spine showed preserved alignment and vertebral morphology with a patent canal and no fractures, destructive lesions, or extradural masses (Figure 1).

**Figure 2:**
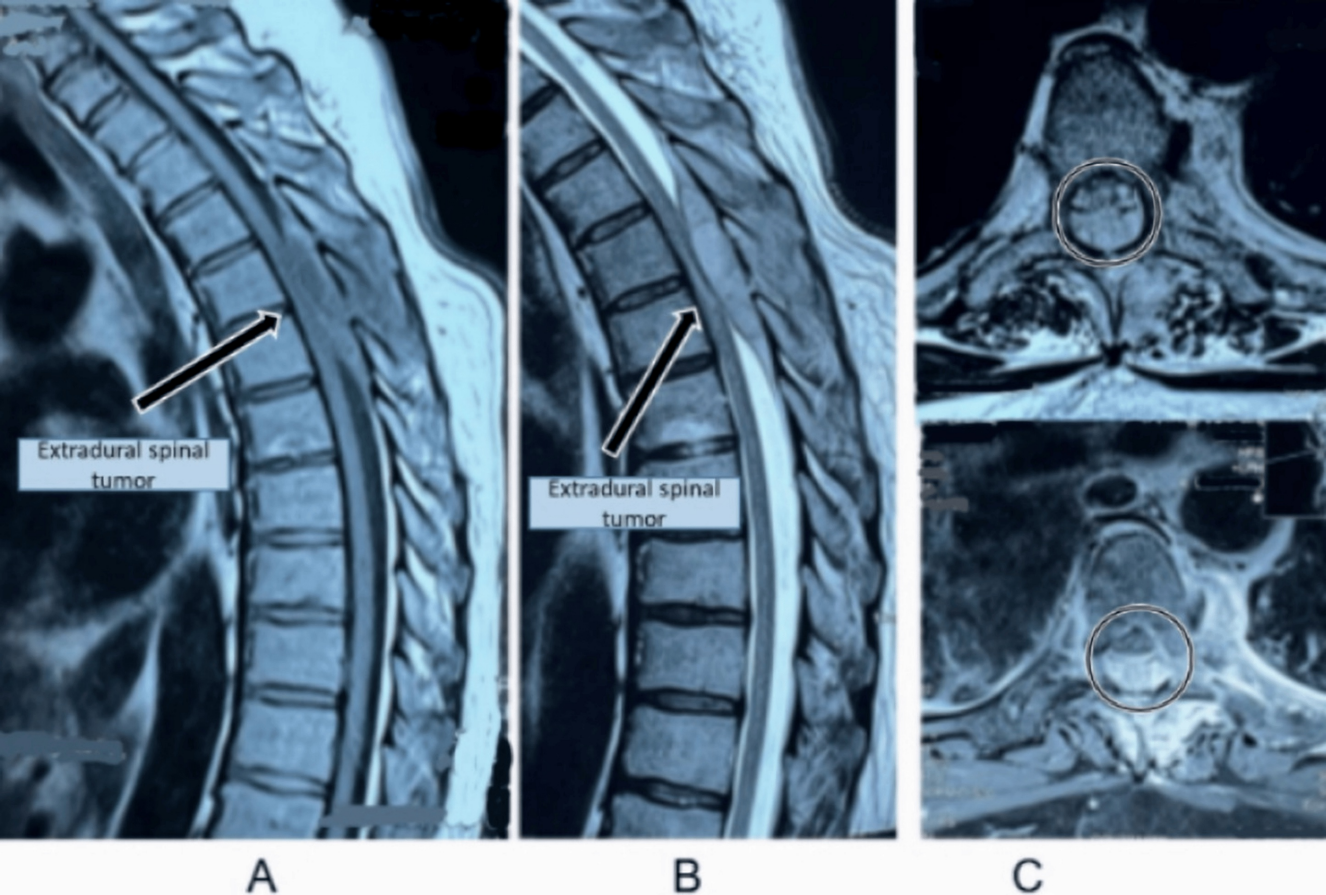
Magnetic resonance imaging of the thoracic spine with gadolinium contrast. (A) Sagittal T1-weighted post-contrast image shows an enhancing extradural spinal tumor (arrow) located between T3 and T6, displacing the spinal cord anteriorly. (B) Sagittal T1-weighted post-contrast sequence confirms the extradural lesion at the mid-thoracic level (T3–T6) with marked mass effect over the spinal cord. (C) Axial T1-weighted post-contrast image at the level of T4–T5 demonstrates the tumor within the spinal canal (circled), producing compression of the dural sac and spinal cord.

A hematologic/oncologic work-up was undertaken to exclude systemic malignancy (metastasis/lymphoma) and infectious etiologies; no systemic source was identified. Gadolinium-enhanced MRI of the thoracic spine revealed a posterior extradural mass extending from T3 to T6, producing significant cord compression and associated hematomyelia (Figure 2). Axial and sagittal sequences demonstrated a heterogeneous extradural lesion with minimal contrast enhancement and marked anterior displacement of the cord.

**Figure 3:**
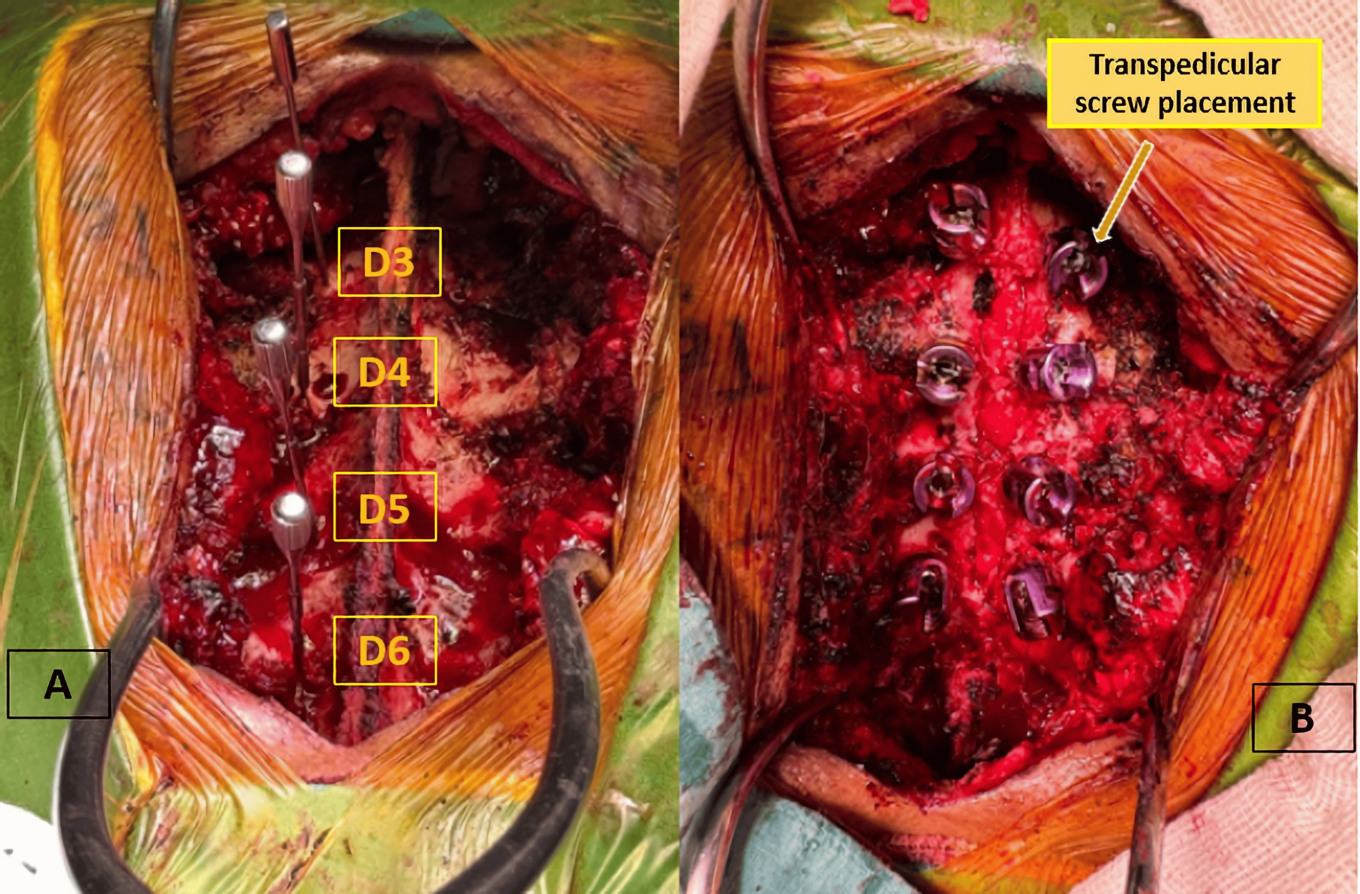
Intraoperative images of transpedicular screw placement in the thoracic spine. (A) Exposure of the thoracic spine with identification of vertebral levels D3–D6 and preparation of the pedicles for screw insertion. (B) Final intraoperative view showing correct placement of transpedicular screws at D3–D6, achieving stabilization of the thoracic spine after tumor resection.

After informed consent, surgery was performed under general anesthesia in the prone position. A posterior approach with wide subperiosteal exposure from T3 to T6 allowed transpedicular screw placement for intraoperative stability (Figure 3), followed by wide laminectomy (T3-T6) and gross-total resection of the posterior extradural mass with adequate decompression of the spinal cord.

**Figure 4:**
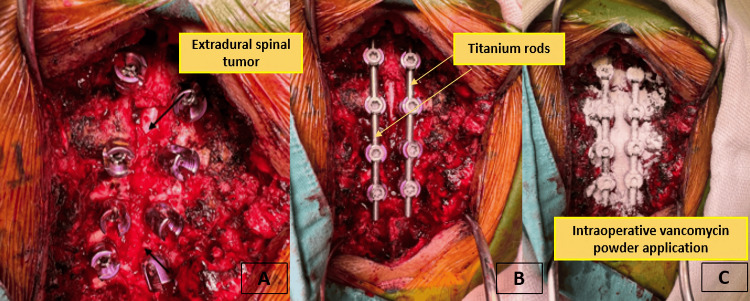
Intraoperative views during thoracic spine stabilization. (A) Identification of an extradural spinal tumor after exposure of the posterior thoracic elements and placement of pedicle screws. (B) Stabilization achieved with bilateral titanium rods secured to the transpedicular screws. (C) Final intraoperative step with topical application of vancomycin powder over the instrumentation site for infection prophylaxis.

Instrumented fixation with titanium rods was completed, and topical vancomycin powder was applied for infection prophylaxis (Figure 4).

**Figure 5:**
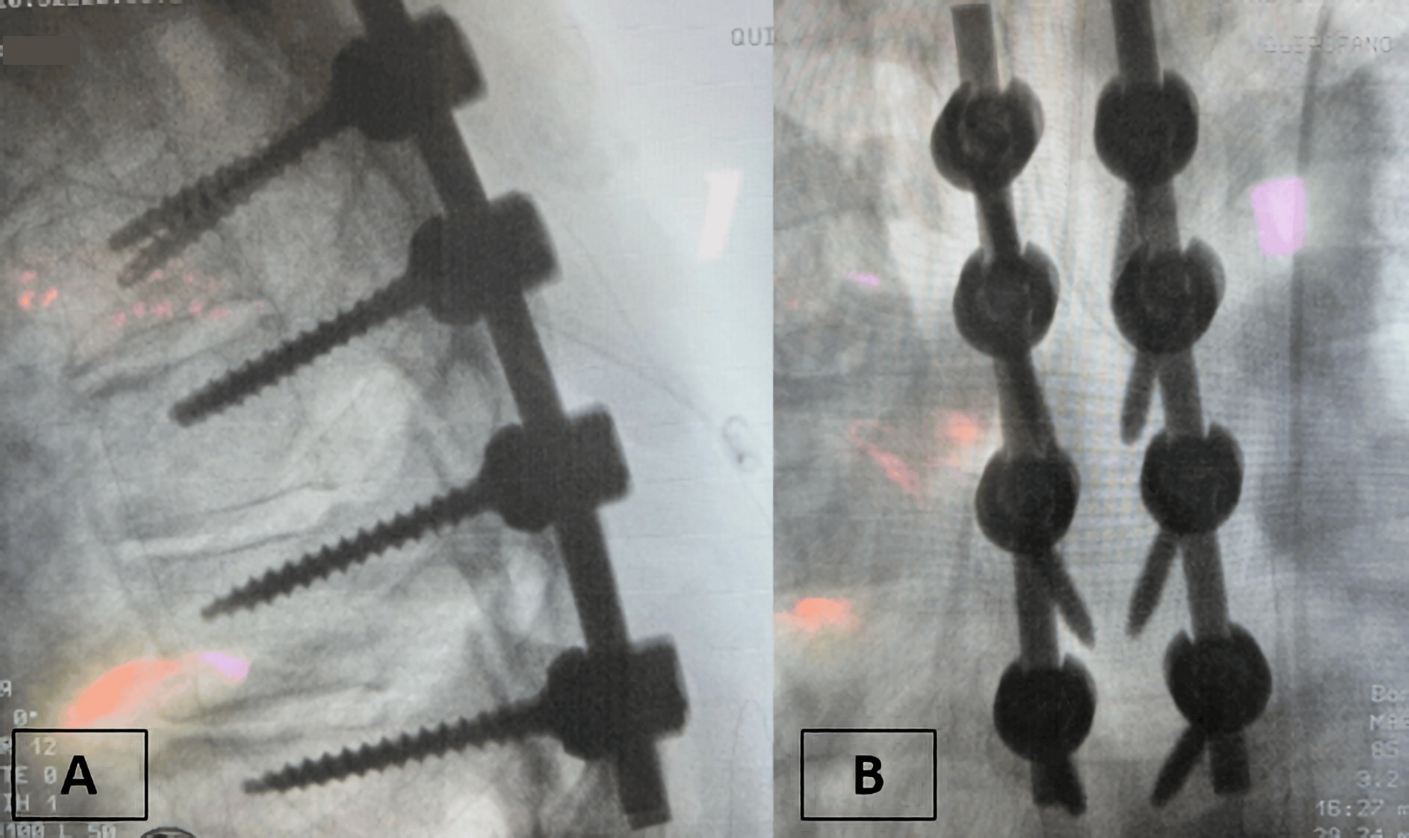
Postoperative radiographs of thoracic spine instrumentation. (A) Lateral view showing correct placement of transpedicular screws and titanium rods providing stabilization of the thoracic spine. (B) Anteroposterior view confirming symmetrical alignment of transpedicular screws and rods across the instrumented levels.

Hemostasis was meticulous, and layered closure was performed; postoperative radiographs confirmed correct instrumentation (Figure 5).

Histopathology (soft-tissue specimen ~50 × 41 mm) showed a well-circumscribed fibroadipose lesion composed of lobules of mature adipocytes separated by dense collagenous stroma, consistent with fibrolipoma. Focal adiponecrosis with foamy macrophages, multinucleated giant cells, and a chronic inflammatory infiltrate (lymphocytes and histiocytes) was present. At the periphery, partially devitalized bone exhibited empty osteocytic lacunae, intertrabecular fibrosis, and reactive angiogenesis, indicating partial bone necrosis with early reparative changes. No cytologic atypia, abnormal vascular proliferation, or increased mitotic activity was identified; no malignancy.

Outcomes and follow-up. At two months, the patient recovered superficial sensation in the lower limbs and sphincter control, indicating conversion from a complete to an incomplete spinal cord injury (sacral function present). At six months, neurological status improved to AIS C, with Medical Research Council (MRC) muscle strength 3/5 at the hips and knees and 2/5 at the ankles. Voluntary lower-limb movement was present but limited by rapid fatigability. Ambulation required orthosis and a walker; he remained unable to maintain an upright position without external support. This partial yet clinically meaningful recovery was achieved through surgical decompression/stabilization and an intensive physiotherapy program (three supervised sessions per week), focused on lower-limb isometric strengthening, postural re-education, assisted gait training with orthosis, and functional electrical stimulation to promote voluntary muscle contraction.

## Discussion

Spinal epidural lipomatous lesions represent a heterogeneous group of benign entities, including simple lipomas, angiolipomas, and fibrolipomas, all capable of producing clinically significant spinal cord compression. Although uncommon, these lesions pose a diagnostic challenge because they often mimic infiltrative or metastatic processes on imaging. Lu et al. (2024) conducted one of the most comprehensive analyses of spinal epidural angiolipomas to date, reporting that 63% of cases were located in the thoracic region, with a female predominance (1:1.4) and a mean diagnostic age of 49 years [4]. Gross-total resection was associated with excellent postoperative neurological outcomes. Similar findings were described by Abdelhameed et al. (2024) [5] and Singh et al. (2023) [6], in which thoracic epidural angiolipomas caused progressive paraparesis and demonstrated marked clinical improvement following surgical decompression.

Taken together, these studies reveal a consistent demographic pattern, showing a predominance among postmenopausal women aged 47-74 years. This trend supports the hypothesis that hormonal changes, particularly the decline in estrogen, may influence adipose tissue remodeling or vascular proliferation, thereby promoting lesion expansion and symptom onset in this population [4,6].

A more recent report by Khanna et al. (2025) described an extradural spinal fibrolipoma in a child that caused significant spinal cord compression and showed favorable postoperative recovery [7]. Although the metabolic and physiological contexts differ between pediatric and adult patients, this case confirms that fibrolipoma represents a well-defined histopathological entity within the epidural compartment, capable of reproducing the clinical and radiological features of other lipomatous lesions such as angiolipoma.

In contrast to these previously reported cases, our patient was a middle-aged man with type 2 diabetes mellitus who presented with a thoracic extradural mass extending from T3 to T6, accompanied by progressive paraplegia. Histopathologic examination revealed mature adipose tissue interlaced with dense fibrous stroma, consistent with fibrolipoma, along with adiponecrosis, devitalized bone, and chronic inflammatory infiltrate, but notably without neoplastic atypia. These findings indicate a benign fibrofatty proliferation exhibiting secondary ischemic and inflammatory changes, rather than a true neoplastic process. Chronic microangiopathy and tissue hypoxia, hallmarks of diabetes, likely predisposed the epidural adipose tissue to degenerative or necrotic alterations.

This interpretation aligns with the findings of Lotan et al. (2020), who reported spinal epidural lipomatosis associated with progressive myelopathy in non-obese patients with type 1 diabetes mellitus, attributing the pathogenesis to metabolic dysregulation and microvascular dysfunction, which promote abnormal accumulation or degeneration of epidural fat and subsequent spinal cord compression [8]. Collectively, these observations suggest that while hormonal modulation may explain the predominance of epidural lipomatous lesions in postmenopausal women, metabolic-vascular mechanisms inherent to diabetes could give rise to fibrofatty pseudotumoral lesions that mimic the clinical and radiological behavior of true lipomatous tumors.

The favorable clinical trajectory restoration of superficial sensation within two months and partial remission at six months reinforces the effectiveness of prompt surgical decompression and stabilization. Additionally, applying intraoperative vancomycin powder likely contributed to mitigating infection risk in this diabetic patient.

## Conclusions

Extradural spinal tumors, though uncommon, should be considered in patients presenting with persistent dorsal pain and progressive neurological deficits. This case highlights the diagnostic challenge posed by atypical presentations, including associated hematomyelia and the presence of diabetes mellitus as a complicating comorbidity. Early MRI evaluation is essential to achieve accurate diagnosis and timely surgical planning. Prompt decompression and stabilization remain the cornerstone of management, while structured postoperative rehabilitation enables functional recovery even when neurological improvement is partial. Histopathological confirmation of fibrolipoma in this patient underscores the importance of correlating radiologic, surgical, and pathologic findings. Reporting such cases expands the clinical understanding of extradural spinal lesions and reinforces the need for early recognition and multidisciplinary management.
